# Synchrotron XRF and Histological Analyses Identify Damage to Digestive Tract of Uranium NP-Exposed *Daphnia magna*

**DOI:** 10.1021/acs.est.2c07174

**Published:** 2023-01-04

**Authors:** Ian Byrnes, Lisa Magdalena Rossbach, Jakub Jaroszewicz, Daniel Grolimund, Dario Ferreira Sanchez, Miguel A. Gomez-Gonzalez, Gert Nuyts, Estela Reinoso-Maset, Koen Janssens, Brit Salbu, Dag Anders Brede, Ole Christian Lind

**Affiliations:** † Faculty of Environmental Sciences and Natural Resource Management, Norwegian University of Life Sciences, Center for Environmental Radioactivity (CERAD), P.O. Box 5003, 1433 Ås, Norway; ‡ Faculty of Materials Science and Engineering, Warsaw University of Technology, Woloska Street 141, 02-507 Warsaw, Poland; § Swiss Light Source, Paul Scherrer Institute (PSI), 5232 Villigen, Switzerland; ∥ Diamond Light Source Ltd., Harwell Science and Innovation Campus, Didcot, Oxfordshire, OX11 0DE, United Kingdom; ⊥ AXIS Group, NANOlab Center of Excellence, Department of Physics, University of Antwerp, Groenenborgerlaan 171, 2020 Antwerp, Belgium

**Keywords:** X-ray fluorescence, X-ray absorption computed tomography, X-ray absorption spectroscopy, toxicokinetics, nanotoxicology, uranium, nanoparticles

## Abstract

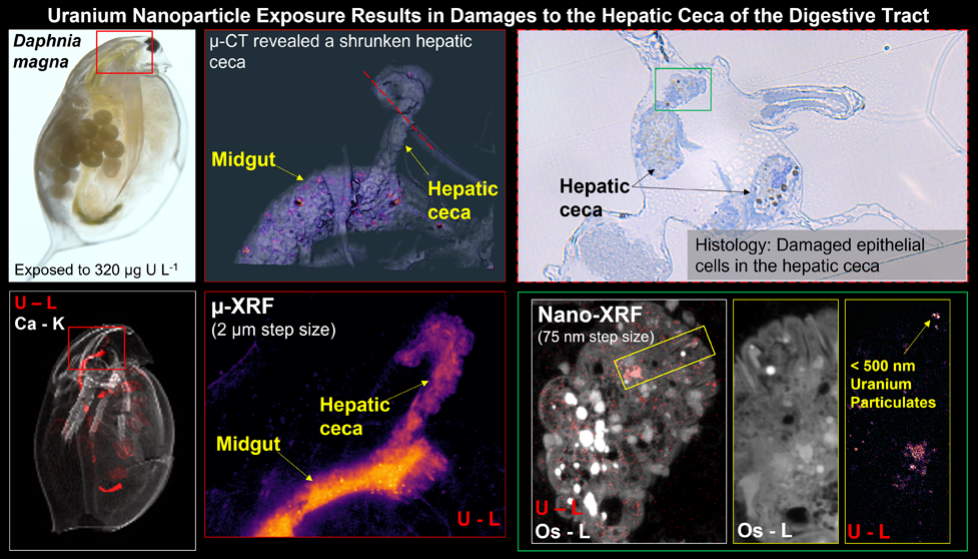

Micro- and nanoscopic X-ray techniques were used to investigate the relationship between uranium (U) tissue distributions and adverse effects to the digestive tract of aquatic model organism *Daphnia magna* following uranium nanoparticle (UNP) exposure. X-ray absorption computed tomography measurements of intact daphnids exposed to sublethal concentrations of UNPs or a U reference solution (U_Ref_) showed adverse morphological changes to the midgut and the hepatic ceca. Histological analyses of exposed organisms revealed a high proportion of abnormal and irregularly shaped intestinal epithelial cells. Disruption of the hepatic ceca and midgut epithelial tissues implied digestive functions and intestinal barriers were compromised. Synchrotron-based micro X-ray fluorescence (XRF) elemental mapping identified U co-localized with morphological changes, with substantial accumulation of U in the lumen as well as in the epithelial tissues. Utilizing high-resolution nano-XRF, 400–1000 nm sized U particulates could be identified throughout the midgut and within hepatic ceca cells, coinciding with tissue damages. The results highlight disruption of intestinal function as an important mode of action of acute U toxicity in *D. magna* and that midgut epithelial cells as well as the hepatic ceca are key target organs.

## Abstract 2

The presented study utilized synchrotron-based micro- and nanoscale elemental imaging techniques to reveal new information on the fate and effects of uranium bioaccumulation in freshwater model organism *Daphnia magna*.

## Introduction

Uranium (U) is released to the environment from a series of naturally occurring U rich minerals and bedrocks such as alum shale^1^ and granite. Uranium is also associated with the release from anthropogenic sources, particularly those stemming from the nuclear weapon and fuel cycles,^2^ such as U mining and milling industries,^3^ nuclear reactor accidents,^4,5^ nuclear weapon detonations,^6^ nuclear waste storage,^7,8^ nuclear fuel reprocessing,^9^ civilian and military use of depleted U,^10^ and, potentially, from the catalyst industry.^11^

In the environment, U can be present in different physicochemical forms varying in size and charge properties. The speciation (i.e., low-molecular-mass (LMM) species, colloids, and particles) is known to influence the mobility and potential transfer of U in the environment, where LMM species (<1 nm) are assumed to be mobile and bioavailable and colloidal forms (1 nm to 0.45 μm) including nanoparticles (NPs) can be relatively mobile, while particles (>0.45 μm) are considered inert.^3,10,12,13^ Molecular growth processes or weathering of minerals and nuclear fuel material may give rise to nanoscale U particles with properties that may differ from those of ions and larger particles with respect to mobility, biological transfer, and toxicity.^14−16^ Uranium concentrations in aquatic systems vary widely depending on the surrounding minerals and sedimentary rock formations as well as anthropogenic activities, in some cases exceeding the World Health Organization (WHO) guideline value (<30 μg U L^–1^)^17^ for drinking water by 2 orders of magnitude.^18,19^ Uranium is especially problematic for aquatic ecosystems where it is known to be taken up into the food web and exhibits a chemotoxicity that can lead to acute effects.^20−22^ The freshwater invertebrate *Daphnia magna* is a preferred model for aquatic toxicological studies due to their role as primary consumers of various algae and bacterial species as well as their functional role in nutrient cycling.^23,24^*Daphnia magna* are highly sensitive to waterborne U where chronic effects have been shown at concentrations > 10 μg L^–1^,^25^ while the 48 h LC_50_ (lethal concentration in 50% of the population) has been reported to occur at concentrations > 390 μg L^–1^,^26^ depending on water conditions such as pH or the presence of U binding ligands.^27^ Traditionally, aquatic toxicology studies have relied on total water concentrations and body burden measurements that lack detailed information to identify underlying toxicokinetic mechanisms. However, a recent study has detailed the biodistribution of internalized U and identified target organs^28^ that include the digestive tract, in agreement with studies that point toward nutrient uptake related to disruption of intestinal processes as a potential toxic mode of action.^26,29^ Upon ingestion of metal species such as metal NPs, the intestine presents a highly exposed organ as well as the primary barrier for uptake.^30^ Furthermore, colloids such as NPs inherit distinct properties from LMM species including the ability to cross biological membranes and accumulate in tissues resulting in heterogeneous biodistributions.^31^ Therefore, spatial distribution and characterization of U species within the digestive tract can provide insights into the toxicokinetic mechanisms underpinning acute effects from the exposure, especially when paired with histological analyses of tissues with cell damage.

Micro- and nano-focused X-ray spectroscopic methods, including X-ray fluorescence (XRF) mapping, are powerful tools for investigating the spatial distributions of metal NPs down to the organ, tissue, and cell level.^32^ In *D. magna*, elemental distribution studies have identified metal accumulation in the intestine, but have so far not differentiated between various compartments or phases, such as luminal contents versus epithelial cells.^33−35^ However, recent synchrotron beamline advances have improved the resolution and detection limits such that the distribution of metals associated with tissues and cells may be identified.^28,36,37^

The objectives of the current study were to characterize uptake and biodistribution in *D. magna* exposed to engineered uranium nanoparticles (UNPs) or a U reference solution (U_Ref_) aiming to identify target organs and tissues related to adverse effects observed in the digestive tract. To this end, micro- and nano-focused, synchrotron-based XRF elemental mapping, with anatomical and histological analyses, was used to assess whether biological effects at the organ and tissue levels were co-localized with U.

## Materials and Methods

### Uranium Nanoparticles

Uranium nanoparticles were synthesized using a natural U source.^38,39^ Particles were stored as lyophilized, dry powder aliquots in a N_2_-purged bottle, inside a desiccator at room temperature. Suspensions (1.0 g U L^–1^) of UNPs were prepared immediately prior to exposure (Supporting Material, Section S1). All UNP stock solutions were characterized for individual particle size, aggregation state, and surface charge using transmission electron microscopy (TEM) and dynamic light scattering (DLS), which is described in further detail in the Supporting Material (Section S1).

### *Daphnia magna* Exposure Experiment

Laboratory-cultured, adult (<7 days) *D. magna*, DHI strain (DHI Water & Environment, Hørsholm, Denmark), were exposed in moderately hard reconstituted water (MHRW, pH 6.8) for 48 h at sublethal concentrations, 320 ± 31 μg U L^–1^ UNP and 159 ± 14 μg U L^–1^ U_Ref_, based on LC_50_ values determined in a previous study.^28^ The U_Ref_ solution was prepared from a U oxide standard (1.0 g L^–1^ in 2% HNO_3_; CRM 129-A, US Department of Energy, Argonne, Illinois). All exposed daphnids were removed from normal culturing conditions, including feed, 24 h prior to the start of the experiment to clear their digestive tract as much as feasible. Size fractionation measurements were conducted to assess the LMM (<3 kDa), colloidal (3 kDa < *x* < 0.45 μm), and particulate (>0.45 μm) fractions (Supporting Material, Section S1). After 48 h, daphnids (*n* = 3) were prepared for whole-body burden (ng U daphnid^–1^) measurements using inductively coupled plasma mass spectrometry (ICP-MS, Agilent 8900, Mississauga, California).

### X-ray Absorption Computed Tomography and Microscale X-ray Fluorescence Imaging

Individual *D. magna* specimens for computed tomography (CT) scanning were placed in a fixative solution of 2.5% glutaraldehyde and 3% paraformaldehyde in a 0.1 M Na cacodylate buffer at 4 °C overnight. Next, the samples were washed in fresh 0.1 M Na cacodylate buffer and dehydrated through a graded ethanol series (30, 50, 70, 90, 95% 1 × 60 min, and 100% 2 × 30 min). Dehydrated, suspended samples were stored at 4 °C until measurement by CT using an XRadia MicroXCT-400 (Carl Zeiss AG, Oberkochen, Germany). Daphnid samples, secured inside an Eppendorf tube, were rotated 360° along their central axis and 1000 tomographic projections were collected per sample at a 2 μm pixel resolution. Volumetric rendering (2 μm^3^ voxel size) of the results was completed using Bruker visualization software solutions (CTVOX, CTVOL, CTAN, Bruker Nano GmbH, Berlin, Germany).

Preserved, whole organisms were prepared for synchrotron-based micro-XRF (μ-SRXRF) by fixation in 5% methanol for 10 min followed by dehydration by graded acetone series (70, 80, 90% 1 × 10 min, 98, and 100% 2 × 10 min) and submersion in 2 mL of hexamethyldisilazane (HMDS) for 1 h.^28^ Subsequently, 1.8 mL of HMDS was carefully removed, and samples were dried overnight in a desiccator with an applied vacuum of 200 mbar. These preserved samples were stored in Eppendorf tubes and kept at room temperature until measurement.

High-sensitivity μ-SRXRF scanning of preserved specimens was conducted at the microXAS beamline (X05LA) at the Swiss Light Source (Paul Scherrer Institute, SLS, Switzerland). Organisms were secured on the sample holder by either Kapton tape or by gluing to the end of a wooden toothpick. Whole-body scans were collected using 20 μm step size and 200 ms dwell time followed by high-resolution scanning (2 μm step size, 200 ms dwell time) of a selected region of interest (ROI) of the *D. magna* digestive tract. A 17.2 keV incident beam was focused using a Kirkpatrick–Baez (KB) mirror system to a size of 1 μm^2^, and the sample was raster-scanned in projection mode. A photon flux of 2 × 10^10^ ph s^–1^ was obtained. X-ray fluorescence spectra were collected using four silicon drift detectors (SDD; Ketek GmbH, Germany) positioned around the sample at 50° to the incoming beam. The results were fitted using PyMCA and elemental maps were compiled and colored with ImageJ (Figure S1A).^40,41^

Moreover, U L_III_-edge micro X-ray absorption near-edge structure (μ-XANES) spectra were collected on points within the ROI of the daphnid in fluorescence and transmission mode. Multiple spectra (*n* = 9) were collected in 1 eV increments from ∼100 eV below the U L_III_-edge (17.163 keV) to ∼300 eV above. Processing of the μ-XANES spectra was conducted using the ATHENA software^42^ and qualitatively compared with μ-XANES spectra of UNP dry powders and reference UO_2_ and U_3_O_8_ spectra (UO_2_, U_3_O_8_, Institute of Energy Technology, Kjeller, Norway) that were measured at HASYLAB, beamline L (unpublished data).

### Combined Histological Analysis and Synchrotron-Based Nanoscale X-ray Fluorescence Imaging

Sections of *D. magna* samples were prepared for histological analysis and synchrotron-based nano-XRF (nano-SRXRF). In brief, whole organisms were subjected to overnight fixation (2.5% glutaraldehyde and 3% paraformaldehyde in a 0.1 M Na cacodylate buffer, pH 7.2) at 4 °C. The following day, the samples were washed in fresh buffer and decalcified in 10% HCl for 30 min followed by a 1% osmium (Os) tetroxide buffer stabilization for 1 h in the dark at constant shaking. Next, the samples were washed in fresh buffer again and dehydrated in a graded ethanol series (30, 50, 70, 90% 1 × 1 h, and 100% 3 × 1 h) before embedding in EPON resin (Agar Scientific Ltd., Essex, United Kingdom).

Sections of 1–5 μm (histology) and 1 μm (nano-SRXRF) were cut using an ultramicrotome equipped with a diamond knife (Diatome Ltd., Nidau, Switzerland). Histological sections were dried on a glass slide and stained with Stevenell Blue dye. Sections were imaged at 10×, 20×, 40×, and 100× magnifications on a Leica DM6B light microscope using the LAS X analysis software (Leica Microsystems, Wetzlar, Germany).

Sections for nano-SRXRF were mounted on 5 × 5 mm^2^ SiN_3_ membranes (Silson Ltd., Warwickshire, U.K.). X-ray fluorescence scanning was carried out at the I14 Hard X-ray Nanoprobe beamline (50 nm beam size) of the Diamond Light Source (U.K.)^43^ using an incident beam energy of 17.3 keV and a four-element silicon drift detector (SGX-RaySpec, U.K.). A resulting flux on the sample was approximately 5 × 10^9^ photons s^–1^.^43^ Coarse maps were obtained using a 225 nm step size and a 200 ms dwell time, while 75 nm step size and 400 ms dwell time were used for high-resolution maps. The PyMCA suite was used for batch fitting and primary analysis of map (Figure S1B).^40^ Further image processing was done using the ImageJ software.^41^

Additional analyses of daphnid midgut tissues were conducted using scanning transmission electron microscopy (STEM) with energy-dispersive X-ray spectroscopy (EDS) (described in detail in the Supporting Material, Section S3).

## Results and Discussion

### Nanoparticle and Exposure Media Characterization

Dry UNPs were characterized in a parallel study using TEM, X-ray diffraction analysis, and μ-XANES showing that the NPs were between 3 and 5 nm in diameter and most closely resembled UO_2_ after synthesis but appeared to have oxidized by the time of synchrotron measurements.^28^ The mean size of UNP aggregates in the UNP stock suspension was 185.6 ± 0.6 nm, while the ζ potential was −9.48 mV (Table S1). These results are consistent with Byrnes et al.^28^ and indicate a propensity of the UNPs to aggregate in aqueous suspensions.^44^ This notion was corroborated by size fractionation measurements of the UNP and the U_Ref_ exposure media (MHRW, pH 6.8)^45^ (Figure S2). After 48 h, colloidal and particulate fractions (>3 kDa) were large in both the UNP (62%) and the U_Ref_ (64%) and the LMM fractions were comparable (39% UNP, 36% U_Ref_), leading to similar U species size distributions between both treatments.

### X-ray Absorption Computed Tomography Identified Morphological Effects from Uranium Exposure

**Figure 1:**
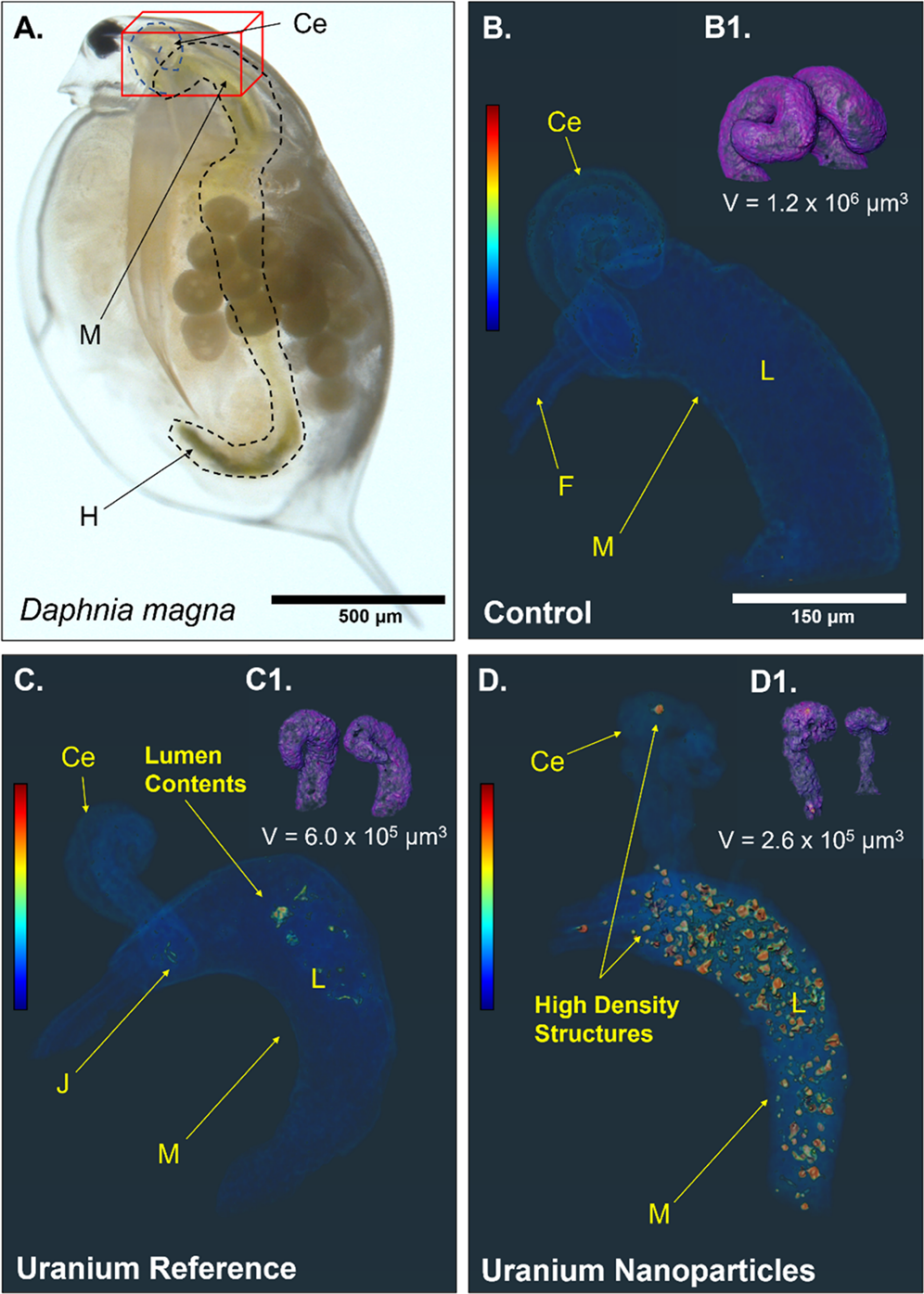
(A) Light microscopy image of *D. magna* showing the midgut, hepatic ceca, and the hindgut (enclosed by the dashed line), and the ROI where the hepatic ceca connect to the midgut (red box). Tomographic renderings (voxel size = 2 μm^3^) of this region are shown for the (B) control, (C) U_Ref_ solution (159 μg U L^–1^), and (D) UNP (320 U μg L^–1^)-exposed organisms. Tomographic analyses provided the volume of the hepatic ceca for the (B1) control, (C1) U_Ref_-, and (D1) UNP-exposed daphnid. Colorbar indicates relative density per tomographic reconstruction and is scaled linearly. Abbreviations: hepatic ceca (Ce), foregut (F), midgut (M), hindgut (H), lumen cavity (L), hepatic ceca–midgut junction (J).

Whole-body CT indicated changes to the morphological structure of the digestive tract in the exposed organism, compared with the control (Figure 1). The hepatic ceca and midgut, regions critical to digestion and nutrient absorption, were therefore digitally isolated and rendered independently of the rest of the organism. The reconstructions revealed residual contents within the midgut of all studied organisms that likely included feed (green algae), despite removing the daphnids from feed conditions 24 h prior to exposure. In the UNP-exposed organisms, these luminal contents had a significantly higher density relative to the soft tissues and lumen contents observed in the U_Ref_ and control organism (Figure 1D), suggesting the presence of aggregated UNPs potentially promoted by the daphnid gut chemistry.^30^ Consistent with a parallel study,^28^ these organisms also exhibited a greater total U body burden on average compared with the U_Ref_-exposed daphnid (Figure S3), further indicating that elevated concentrations of U within the digestive tract mainly constituted UNP aggregates. Tomographic renderings revealed that the hepatic ceca of exposed daphnids appeared severely shrunken and straightened (Figure 1C,D), comparable to observations made following Cd exposure.^46^ Based on CT, the volumes of the hepatic ceca were reduced by a factor of ∼2 (159 ± 14 μg U L^–1^ U_Ref_) and ∼4.6 (320 ± 31 μg U L^–1^ UNP) compared to the control. In the UNP-exposed organism, high-density structures, suggesting aggregates, appeared far into the ceca, signifying impaired gut barrier functions that would normally isolate contents within the lumen.

### Microscale XRF Investigations Confirm Extensive Intestinal Damages Are Associated with U Accumulation

**Figure 2:**
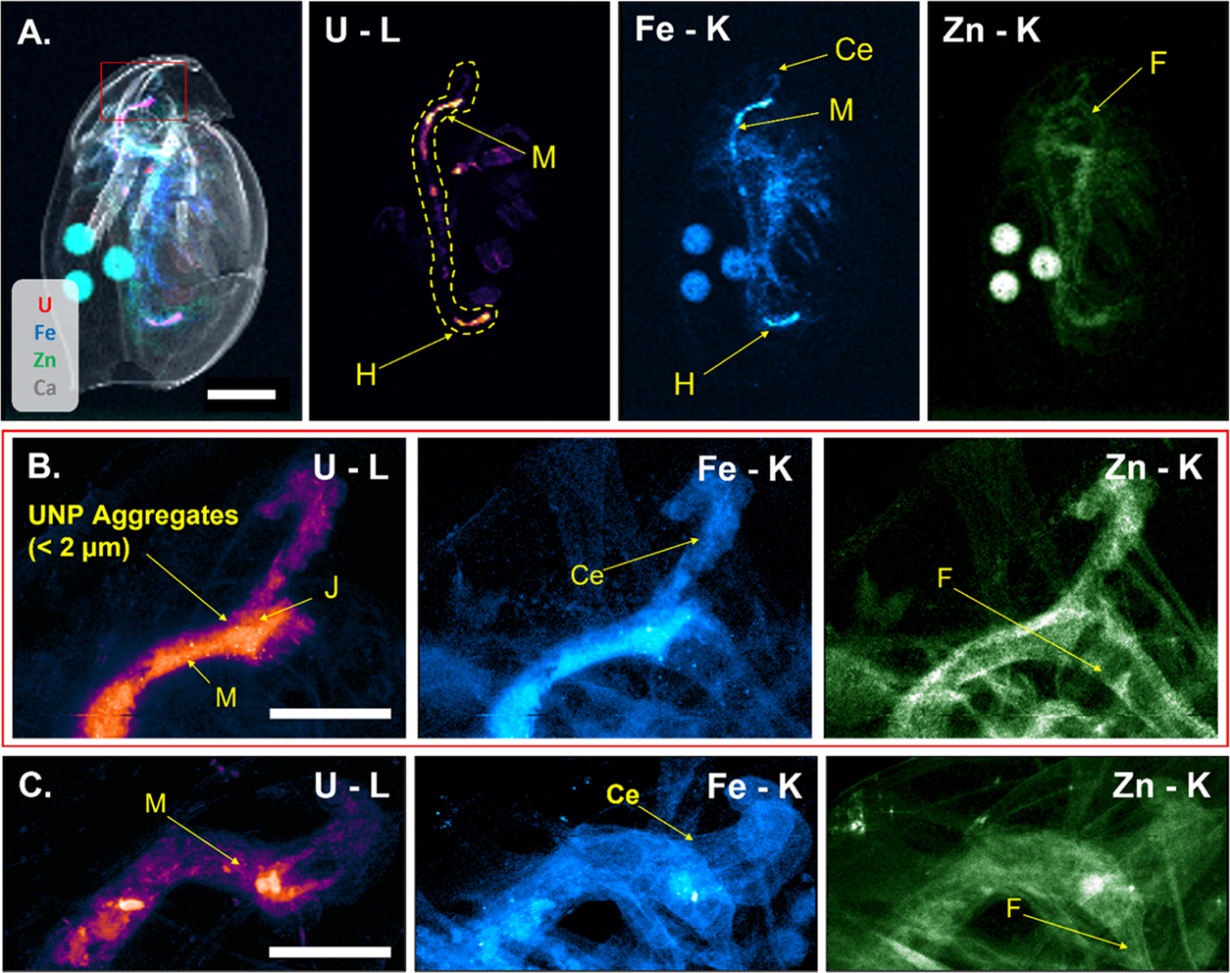
(A) Composite and individual elemental μ-SRXRF mapping (20 μm step size, 200 ms dwell time) of *D. magna* exposed to UNPs (320 μg U L^–1^) showing the whole-body distribution of U (red), Ca (gray), Fe (blue), and Zn (green), and the ROI for high-resolution investigation (red box). The digestive tract is circled by the dashed yellow line. (B) Two-dimensional mapping of the ROI from the same UNP-exposed daphnid via μ-SRXRF (2 μm step size, 200 ms dwell time) and (C) the comparable region studied on a daphnid exposed to the U_Ref_ solution (159 μg U L^–1^). Scale bars represent 500 μm (A) or 100 μm (B, C), and all signal intensities are scaled logarithmically. Abbreviations: hepatic ceca (Ce), midgut (M), hindgut (H), foregut (F), and the hepatic ceca–midgut junction (J).

Low-resolution μ-SRXRF scans of the whole daphnid showed U signals throughout the digestive tract including the hepatic ceca and midgut (Figure 2A). Distributions of Fe and Zn constituted the major elements of the soft tissues, while Ca was indicative of the carapace. Within the digestive tract, a high U signal was also observed within the hindgut. This region is protected by a 1–2 μm cuticle and is associated only with the movement of food and gut material and not with nutrient uptake or digestion;^47^ therefore, it is not necessarily critically affected by U retention. High-resolution mapping of the ROI around the junction of the hepatic ceca and midgut allowed distinguishing between the luminal contents and the epithelial tissues of the organs (Figure 2B). In all imaged daphnids, elevated levels of U were detected at this junction, where the ceca are excreting digestive enzymes into the midgut and the peritrophic membrane is secreted around the food bolus.^23,48^ Uranium translocation from the intestinal lumen to the hepatic ceca was evident, which implies failure of the protective intestinal barrier functions (i.e., peritrophic membrane) that would otherwise prevent ingested materials from entering the ceca. Cellular uptake of U in epithelial tissues was weakly visible due to a low relative intensity compared with the high signals observed in the lumen, where U was strongly associated with gut materials not cleared by the daphnid prior to the exposure (shown in more detail in Figure 5). Finally, U was not observed in the foregut, a region which also bears a 1–2 μm cuticle,^49^ indicating that the retention of U was negligible prior to entry into the midgut.

The μ-XANES spectra on locations of high U intensity in the hepatic ceca and midgut shared the same characteristics as those collected as part of dry UNP characterization work,^28^ suggesting the UNPs retained in daphnids are also oxidized (Figure S4). However, contributing factors from the sample preparation or potential photooxidation incurred on the beamline^50^ could not be excluded and further analysis is required to confirm the results from this work.

### Combined Histological and Nano-SRXRF Analyses of the Hepatic Ceca

**Figure 3:**
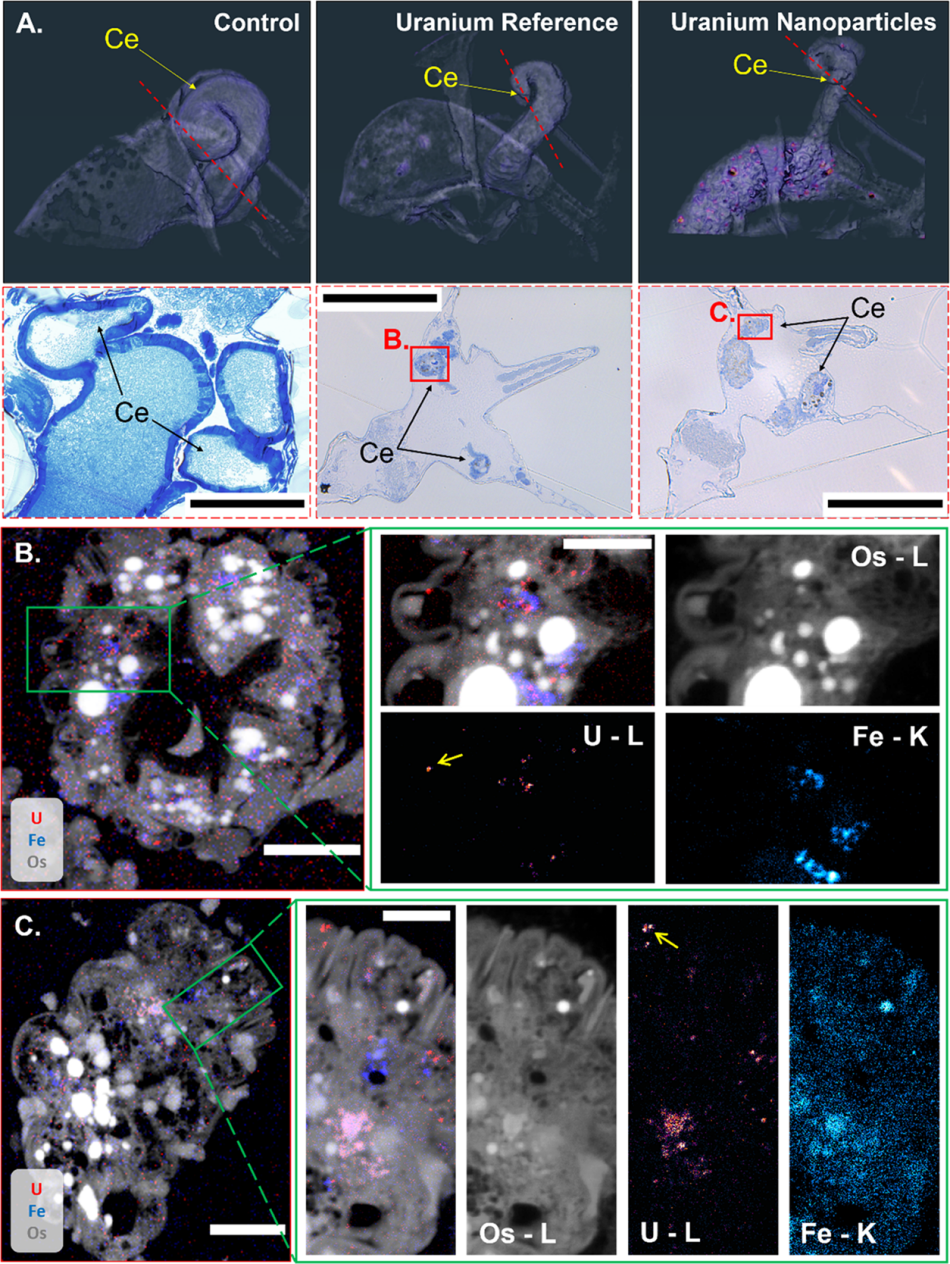
(A) Tomographic reconstruction (top images) of the *D. magna* hepatic ceca (CE) in an unexposed individual (left) and in individuals exposed to 159 μg U L^–1^ U_Ref_ (center) and 320 μg U L^–1^ UNP (right). The location of the histology sections (bottom images) is indicated by red dotted lines, and the areas of hepatic ceca tissues investigated by nano-SRXRF (225 nm step size, 400 ms dwell time) by red boxes. (B, C) Combined U, Fe, and Os nano-SRXRF maps of exposed daphnids (U_Ref_ in B, UNP in C) with green boxes of the ceca tissue region indicating the ROI investigated by high-resolution nano-SRXRF maps (75 nm step size, 400 ms dwell time). The yellow arrows in the U maps indicate one (B) and two (C) U particulates of ca. 560 and 450 nm in size, respectively. Scale bars represent 100 μm (A) and 5 μm (B, C) and all intensities are scaled logarithmically.

Histological analysis of the hepatic ceca showed that the epithelial cells were largely destroyed, with remnants entering the luminal space (Figure 3). In contrast, the control organism exhibited healthy, cuboidal cells that were lined with microvilli. The tissues of the U_Ref_-exposed organism retained some normal structure (Figure 3B) with reduced microvilli present, while such cell features were absent in the UNP-exposed daphnid (Figure 3C). The cell and tissue damage in the hepatic ceca observed in the exposed daphnids was commensurate with the observed reduced organ size and straightening in the CT renderings (Figure 1), indicating that cell damage could be a leading cause of the morphological changes.

Using the Os distribution to align the elemental maps with the histology section, nano-SRXRF scans (Figure 3B,C) showed the presence of U-containing materials throughout the damaged hepatic ceca tissues, further confirming translocation into the organ from the midgut. Both exposures resulted in small (<500 nm) U hotspots distributed throughout the investigated section of hepatic ceca. In UNP-exposed samples, these hotspots were likely small aggregates of UNPs, while, in the U_Ref_-derived organism, these particulates probably originated from the particulate (>0.45 μm) fraction and/or due to aggregation of colloids (Figure S1). Nanoparticles and colloids have the potential to act as diffuse sources of long-term release of ions when embedded in tissues, as observed here in the hepatic ceca, potentially leading to localized stress to cells.^44^ Given the damage to the cell structures and the presence of U throughout the tissues, it is conceivable that hepatic ceca dysfunction is a key event leading to acute mortality observed in toxicity assessments.^28^

### Combined Histological and Nano-SRXRF Analyses of the Midgut

**Figure 4:**
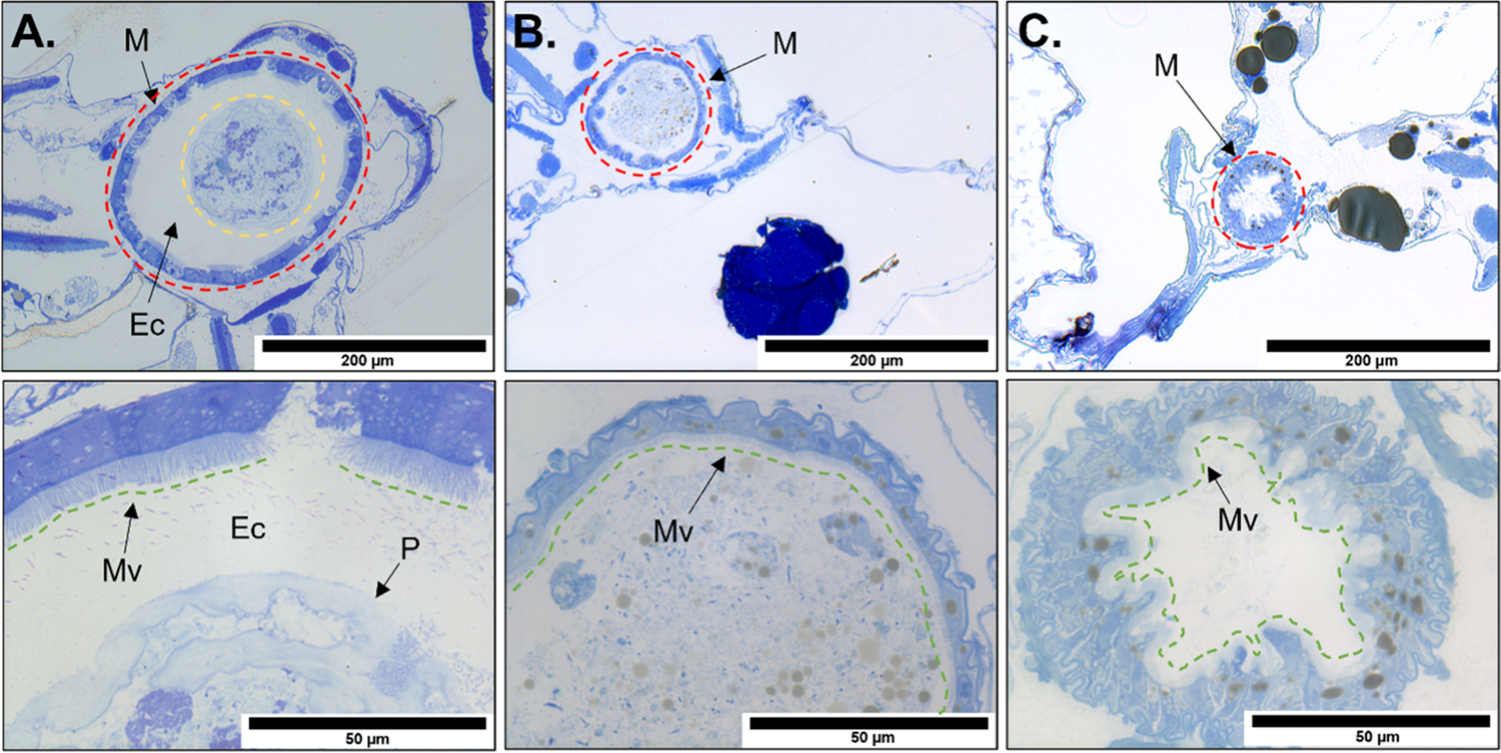
(Top) Histological sections of *D. magna* midgut and surrounding area in a control organism (A), a U_Ref_-exposed organism (159 μg U L^–1^, (B)), and a UNP-exposed organism (320 μg U L^–1^, (C)). The midgut is represented by dashed red lines and, only in the control organism, the lumen contents were confined within the peritrophic membrane are outlined with yellow dashes. (Bottom) High-magnification (100×) images of the epithelial cell wall (green dashed lines) in a control organism (A), a U_Ref_-exposed organism, and a UNP-exposed organism. Abbreviations: midgut (M), ectoperitrophic space (Ec), peritrophic membrane (P), microvilli (Mv).

Histological sections of both UNP and the U_Ref_-exposed *D. magna* revealed intestinal damage and cell distortion in gut epithelia (Figure 4). In UNP-exposed organisms, epithelial cells appeared irregular and protruded into the gut lumen with dilatation of the intercellular spaces and microvilli were damaged. Similar effects, although less pronounced, were observed in the U_Ref_-exposed daphnid that featured lower body burden than the UNP-exposed organisms, indicating that intestinal cell damage could be U-concentration-dependent. These observations are consistent with a previous study of U toxicity to *D. magna* that reported similar damages to intestinal cells.^29^ The peritrophic membrane, a chitinous mesh that confines the lumen contents,^47^ appeared disintegrated in both exposed daphnids with very little ectoperitrophic space remaining between the gut materials and the microvilli (Figure 4). A normally functioning peritrophic membrane was expected to prevent the majority of the UNPs from reaching areas around the epithelial cells as hydrodynamic diameter measurements indicated average UNP aggregate sizes > 130 nm, i.e., larger than the approximate mesh size of the membrane.^48^ Using STEM-EDS to examine the midgut of UNP-exposed organisms, U aggregates were observed around the intestinal epithelia and between the microvilli further indicating that the peritrophic membrane was not functioning (Figure S5). These results are similar to those of Heinlaan et al.,^51^ who observed the absence of the peritrophic membrane after a 48 h exposure to CuO NPs with aggregates spread into the brush border of the epithelial cells. Although the specific mechanisms that lead to peritrophic membrane failure remain unknown, it is conceivable that U-induced stress compromised the function of the epithelial cells that synthesize and secrete the peritrophic membrane.

**Figure 5:**
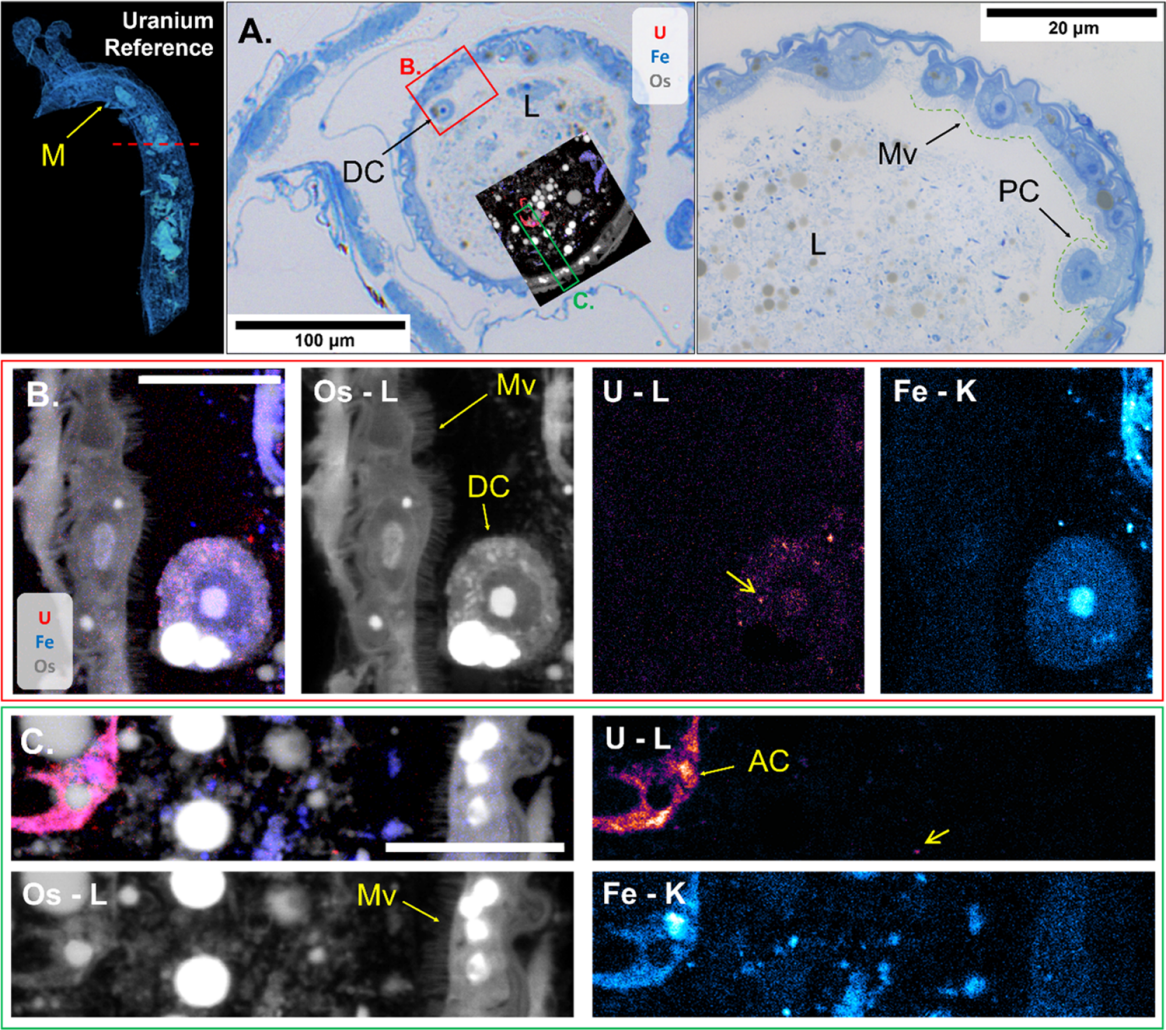
Elemental analysis of histological section from *D. magna* (U_Ref_, 159 U μg L^–1^) showing the U (red) and Fe (blue) distributions using the Os (gray) to orient the features. (A) Histological sections of exposed organism midgut with cell features and areas of nano-SRXRF analysis (area B indicated by a red box and area C indicated by a yellow box). The tomographic rendering (left) shows the approximate location of the section (red dashed line). (B) Detached epithelial cells with small (<500 nm) particulates indicated by the yellow arrow (∼530 nm). (C) Lumen contents including a green algae cell and small (<500 nm) U particulates indicated by the yellow arrow (∼380 nm). Scale bars in (B) and (C) represent 10 μm. All signal intensities are scaled logarithmically. Abbreviations: midgut (M), detached epithelial cell (DC), lumen (L), microvilli (Mv), protruded epithelial cell (PC), algae cell (AC).

High-resolution nano-SRXRF mapping of a dorsal midgut section of U_Ref_-exposed daphnid was used to show the localization of U between the lumen and the epithelial cells (Figure 5). A large area of the lumen and epithelial wall was selected for study and overlaid with the histological section using the Os map to align the images. Within the lumen, U particulates (<500 nm) were prevalent, while U was also associated with a detached epithelial cell and partially digested algae content. The small, ca. 300–600 μm sized particulates of U observed in the lumen (Figure 5B,C) were similar to those within the hepatic ceca (Figure 3B). The μ-SRXRF results (Figure 2B) indicated uptake into the intestinal epithelia. However, any U present in the epithelial cells or the microvilli of the section measured by nano-SRXRF remained below the detection limit. Uranium-bearing precipitates have previously been observed in histological sections of chronically exposed *D. magna*,^29^ but these phenomena were not identified in the region of epithelial cells as presented here. However, the detached epithelial cell contained substantial U signals in the membrane, cytosol, and nucleus. Shedding is normally part of tissue maintenance,^52^ but may be enhanced by stress.^53^ It is thus tempting to speculate that shedding is part of the response to U-induced stress, although further work is needed to confirm this notion.

The greatest U signal in the midgut of the U_Ref_-exposed daphnid was observed in a 10 μm, partially digested algae cell (Figure 5C), *Raphidocelis subcapitata*, which are known to effectively bind bioavailable U species.^54,55^ Although the test organisms were removed from feed prior to exposure, complete evacuation of the intestine did not occur and the presence of the U-bearing algal cell demonstrated the relationship between U uptake and binding to gut contents. This observation highlights inherent constraints of whole-body burden measurements that are not able to differentiate between tissue uptake and intestinally confined U present in the lumen.

Overall, the combined XRF and histological analyses used in this study confirmed the presence of U in damaged tissues of the digestive tract of *D. magna*. The application of nanoscopic XRF enabled the visualization of U internalized in hepatic ceca tissues and intestinal cells. Both the UNPs and the U_Ref_ exposures compromised key functions of the intestine. Breakdown of the midgut epithelia, peritrophic membrane disintegration, and deterioration of the hepatic ceca were identified and likely contributed to the U-induced acute toxicity. Collectively, these results demonstrate the power of synchrotron-based XRF methodology to investigate tissue and cell biodistribution of metals and a wide range of toxicants at nanoscale resolution thus providing an improved basis for environmental impact and risk assessments.

## Supporting Information Available

Nanoparticle synthesis and characterization; the *D. magna* culture and exposure conditions; and additional imaging details; uranium nanoparticle suspension and characterization (Section S1); *Daphnia magna* culture and exposure experiments (Section S2); additional imaging measurements (Section S3); XRF sum spectra (Figure S1); uranium nanoparticle dispersion (Table S1); major elements in UNP stock suspension (Table S2); size distributions (Figure S2); uranium body burden (Figure S3); μ-XANES measurements (Figure S4); and transmission electron microscopy (Figure S5) (PDF)
