# Supplementary material for: Synchrotron XRF and Histological Analyses Identify Damage to Digestive Tract of Uranium NP-Exposed Daphnia magna
Source: Environ Sci Technol. 2023 Jan 4;57(2):1071–9. doi: 10.1021/acs.est.2c07174 (PMC9850915; doi:10.1021/acs.est.2c07174)
Supplement: Supplementary file 1 — es2c07174_si_001.pdf [file es2c07174_si_001.pdf]

Synchrotron XRF and Histological Analyses Identify Damage to Digestive Tract of Uranium NP-Exposed *Daphnia magna*

Ian Byrnes[1*], Lisa Magdalena Rossbach[1], Jakub Jaroszewicz[2], Daniel Grolimund[3], Dario Ferreira Sanchez[3], Miguel A. Gomez-Gonzalez[4], Gert Nuyts[5], Estela Reinoso-Maset[1], Koen Janssens[5], Brit Salbu[1], Dag Anders Brede[1], Ole Christian Lind[1*]

[1] Norwegian University of Life Sciences, Center for Environmental Radioactivity (CERAD), Faculty of Environmental Sciences and Natural Resource Management, P.O. Box 5003, 1433 Ås, Norway

[2] Warsaw University of Technology, Faculty of Materials Science and Engineering, Woloska St. 141, 02-507 Warsaw, Poland

[3] Swiss Light Source, Paul Scherrer Institute (PSI), 5232 Villigen, Switzerland

[4] Diamond Light Source Ltd., Harwell Science and Innovation Campus, Didcot, Oxfordshire OX11 0DE, United Kingdom

[5] University of Antwerp, AXIS Group, NANOlab Center of Excellence, Department of Physics, Groenenborgerlaan 171, 2020 Antwerp, Belgium

*Corresponding Author

Phone: +47-93820876 Fax: +47-64948359

Email Address: ian.byrnes@nmbu.no, ole-christian.lind@nmbu.no

Address: Norwegian University of Life Sciences, Center for Environmental Radioactivity (CERAD), Faculty of Environmental Sciences and Natural Resource Management, P.O. Box 5003, 1433 Ås, Norway

Summary

This document includes the supplementary material supporting the methods (further description) and results (2 tables and 4 figures).

Experimental Method

S1. Uranium Nanoparticle Suspension and Characterization

S2. *Daphnia magna* Culture and Exposure Experiments

S3. Additional Imaging Measurements

Results

Figure S1: XRF Sum Spectra

Table S1: Uranium Nanoparticle Dispersion

Table S2: Major Elements in UNP Stock Suspension

Figure S2: Size Distributions

Figure S3: Uranium Body Burden

Figure S4: µ-XANES Measurements

Figure S5: Transmission Electron Microscopy

Experimental Methods:

S1. Uranium Nanoparticle Suspension and Characterization

Stock suspensions (1.0 g U L^{-1}) were prepared by weighing UNPs in a non-static environment on a microbalance and placed in an empty 20 mL glass vial. A dispersion agent, 1% polyoxyethelene glycerol triolate, was applied directly to the dry particles before the addition of 10 mL N_2 purged (4 h) ddH_2O (15 MΩ cm). Immediately afterwards, the UNP suspension was sonicated for 13 min at a 15 % amplitude using a 400-W Branson Sonifier S-450D (Branson Ultrasonics) equipped with a standard 13 mm disruptor tip (model 101-147-037). All prepared stocks were used immediately following sonication.

Dynamic Light Scattering

Zeta-average hydrodynamic diameter of the UNPs in the stock suspension were determined by dynamic light scattering (DLS) measurements using a Malvern Zetasizer ZS (Malvern Instruments Ltd., Worcestershire, United Kingdom) equipped with a 633 nm laser. Measurements were conducted in triplicate, 5 runs each, with autocorrection functions of 10 s. Electrophoretic mobility was measured and zeta-potentials for the stock suspensions were determined by Smoluchowski approximations.

Transmission Electron Microscopy

High resolution transmission electron microscopy (TEM) with energy dispersive X-ray spectroscopy (EDS) was used to image the UNPs from the stock suspension and to measure the diameter of individual particles. Immediately following sonication, 10 µL of stock suspension was added to a 400-mesh Cu-coated formvar-carbon film (Agar Scientific Ltd., Essex, United Kingdom) and allowed to air dry. Samples were measured at 200 kV accelerating voltage on a JEOL JEM-2100F equipped with a Gatan Porius 200D CCD camera (JEOL Ltd., Tokyo, Japan). Uranium fluorescent X-rays were collected by an Oxford X-Max-80 SDD EDS detector at a 0.23 srad collection angle.

Triple Quadrupole Inductively Coupled Plasma Mass Spectrometry

Uranium concentrations in both the stock suspensions and exposure media were determined by triple quadrupole inductively coupled plasma mass spectrometry

(QQQ-ICP-MS; Agilent 8900, Mississauga, CA). All measurements were completed in triplicate and each sample (100 µL) was mixed with 400 µL of ultrapure HNO_3 and the samples were digested for 48 h before dilution with ddH_2O (15 MΩ cm) to final volume of 10 mL. Measurements of digested daphnids had a limit of detection (LOD) of 0.008 µg ^{238}U L^{-1} and a limit of quantification (LOQ) of 0.026 µg ^{238}U L^{-1}. Measurements of digested water samples for media characterization had a LOD of 0.003 µg ^{238}U L^{-1} and a LOQ of 0.009 µg ^{238}U L^{-1}.

Size Fractionation

To determine the particulate (> 0.45 µm), colloidal (0.45 µm < x > 3 kDa), and LMM (< 3 kDa) fractions, QQQ-ICP-MS analysis of size fractionated exposure media was performed. In each exposure group, 1 mL of media was passed through a pre-conditioned 0.45 µm syringe filter (VWR, Radnor, Pennsylvania, United States) and 100 µL was sampled from the filtrate. Next, 400 µL of the < 0.45 µm filtrate was sampled into a pre-conditioned 3 kDa Amicon cellulose membrane filter (Amicon Millipore, Billerica, MA) and centrifuged at 14,000 *g* for 30 min. From the < 3 kDa filtrate solution, 100 µL was sampled for QQQ-ICP-MS measurement to determine the LMM fraction.

S2. *Daphnia magna* Culture and Exposure Experiments

Laboratory cultured *D. magna*, DHI strain (DHI Water & Environment, Hørsholm, Denmark), were reared at 20°C (± 1°C) with a day-night cycle of 16 h light:8 h darkness in M7 media (OECD 2004). The culture media was renewed three times weekly at which point neonates were removed. Daphnids were fed a diet of concentrated algae (*Raphidocelis subcapitata*) at a rate of 5.25 x 10^{-6} cells day^{-1} $daphnid^{-1}$ for neonates and 2.10 x 10^{-7} cells day^{-1} $daphnid^{-1}$ for adults. Synchronized neonates (< 18 h) derived from the second clutch or later were used for exposure experiments.

Uranium nanoparticle and the U_{Ref} exposures were conducted in US EPA moderately hard reconstituted water (MHRW, pH 6.8, 350 µS cm^{-1}, 20°C), which was prepared a week prior to the exposures (USEPA 2002). Daphnids were exposed in groups of 5 individuals in 25 mL of MHRW. The concentration chosen was reflective of sublethal acute effects determined by acute toxicity tests reported previously (Byrnes et al., unpublished). For UNP exposures, 25 mL of MHRW was added to a 50 mL plastic cup and UNP stock additions were added just prior to the start of exposure. Dissolved U_{Ref}

solutions were prepared by pipetting 50 µL from a 100 mg U L^{-1} dilution of U_{Ref} solution (CRM 129-A, Spectrapure Standards AS, Oslo, Norway) into empty 50 mL plastic exposure cups. The U_{Ref} solutions were evaporated to dryness in order to resolve issue related to the low pH originating from the nitric acid. The dry residuals were redissolved in 25 mL of MHRW exposure solution 24 h prior to exposure start. The pH of the U_{Ref} and UNP exposures were confirmed (6.8 ± 0.1) at the start of the experiment.

S3. Additional Imaging Measurements

Laboratory X-ray Absorption Computed Tomography

The reconstructed output from X-ray absorption tomography contained a stack of tomograms (virtual slices), visualized using DataViewer (Bruker Nano GmbH, Berlin, Germany), that reveal the inner morphology of measured organisms in a greyscale that is correlated to the X-ray attenuation. Volumetric rendering of the results was completed using Bruker visualization software solutions (CTVOX, CTVOL, CTAN, Bruker Nano GmbH, Berlin, Germany). In brief, CTVOX and CTVOL handle the rendering of tomographic data and sample coloring and transparency while CTAN provides density examination, size and structure measurements, and region-of-interest analyses.

Analytical Transmission Electron Microscopy

High magnification, subcellular resolution analyses of daphnid sections was conducted using scanning transmission electron microscopy (STEM) with energy dispersive X-ray spectroscopy (EDS) (JOEL JEM-2100F), with particular focus on the intestine and microvilli of LRwhite embedded organisms. Ultrathin sections (<100 nm) were prepared using an ultramicrotome equipped with a diamond knife (Diatome Ltd., Nidau, Switzerland). Sections were mounted on copper slot grids with a formvar carbon film (EM Resolutions Ldt, Sheffield, UK). Samples were analyzed under the same specifications as previously described for individual particle imaging.

Results:

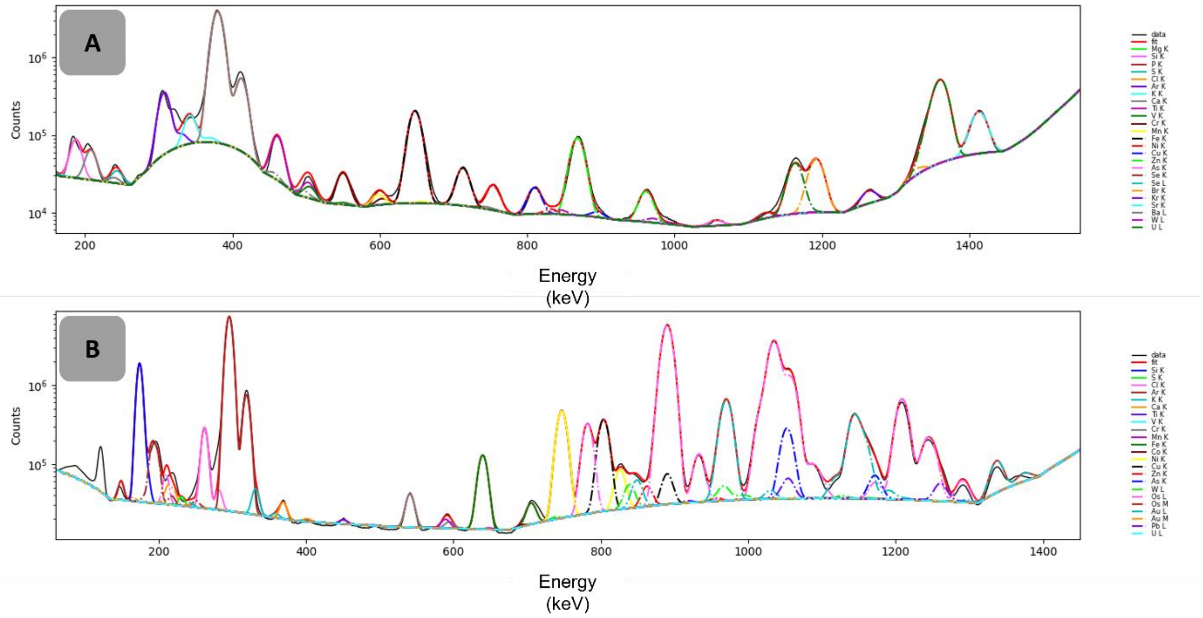

Figure S1: Example XRF sum spectra collected from (A) the hepatic ceca region shown in Fig. 2B obtained at the microXAS beamline and (B) the intestinal cross section shown in Fig. 5C.

Table S1: Uranium Nanoparticle Suspension Characteristics and *D. magna* Exposure Parameters

Experiment	Average Hydrodynamic diameter (nm)	Zeta potential (mV)	Measured UNP Test Concentration 48 h (µg U L^{-1})	Measured U_{Ref} Test Concentrations 48 h (µg U L^{-1})
D. magna Adults (< 7 d)	185.6 ± 0.6	-9.48	320 ± 31	159 ± 14

Table S2: Major constituents of UNP stock suspension measured by QQQ-ICP-MS. Concentrations are reported as average ± 1 standard deviation (n = 3).

UNP Stock Suspension	µg L^{-1}
**Li**	64.4 ± 7.0
**B**	3613 ± 70
**Ti**	800 ± 29
**V**	79.1 ± 1.8
**Mo**	1.5 ± 0.1
**Ag**	28.4 ± 1.5
**Sn**	103 ± 139
**Lu**	1.75 ± 0.02
**U** (mg L^{-1})	639 ± 22

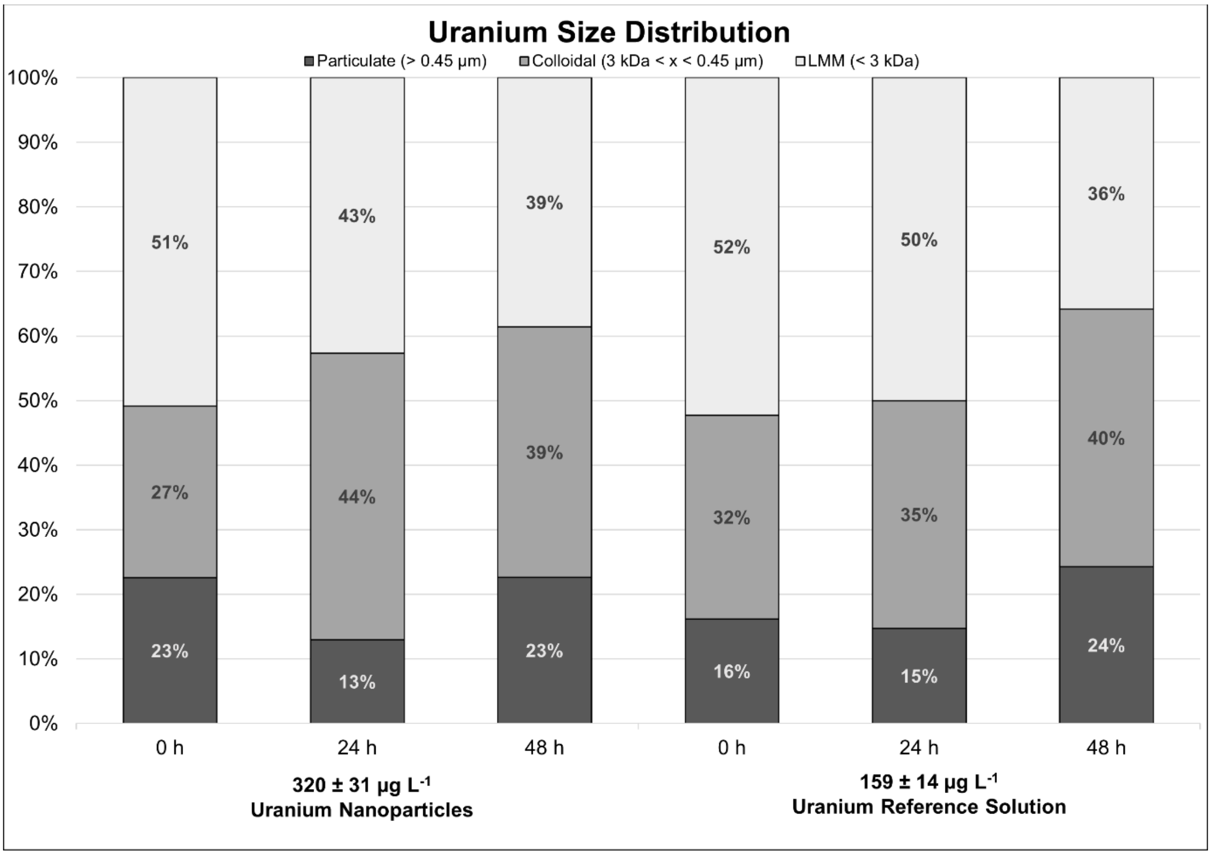

Figure S2: Size distribution of U species in the exposure media for the UNP exposures (*Left*) and U_{Ref} solution exposures (*Right*).

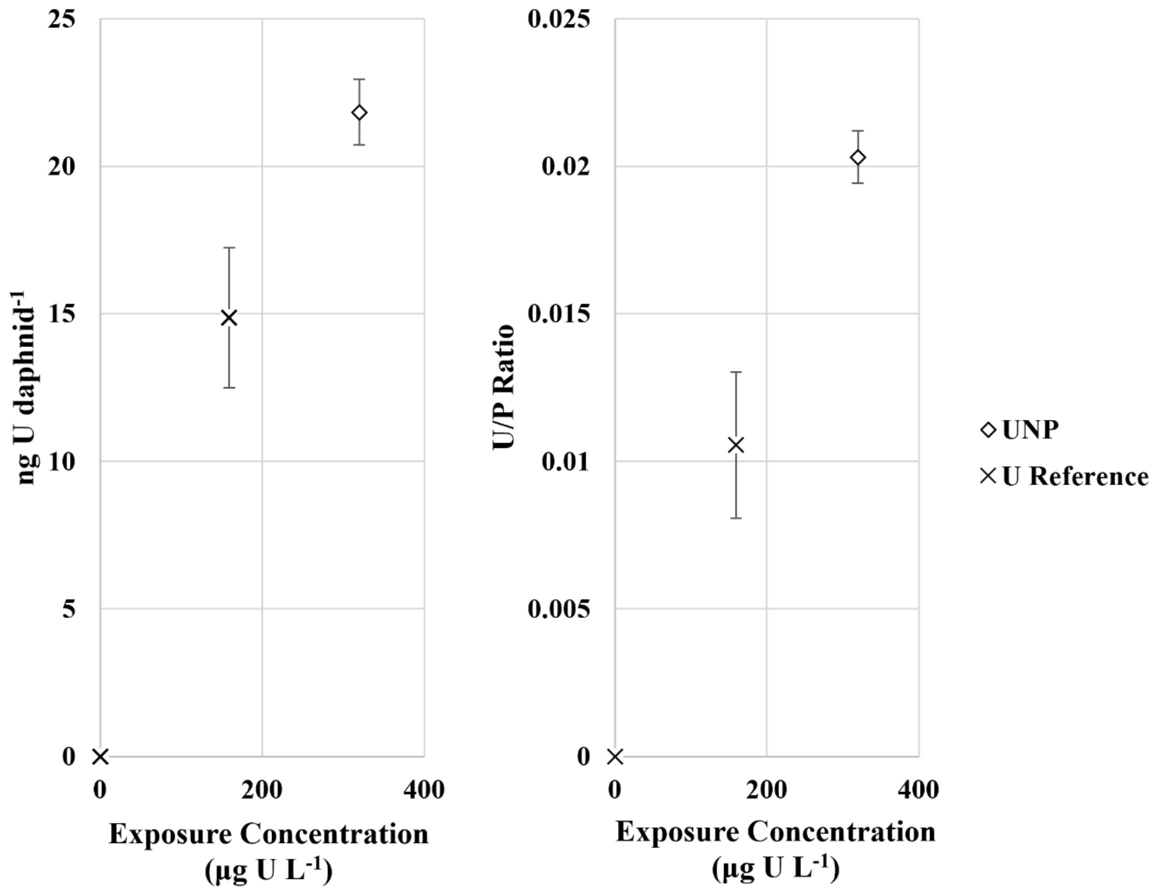

Figure S3: Whole daphnid, U body burden measurements for both the UNP and the U_{Ref} solution exposures based on QQQ-ICP-MS.

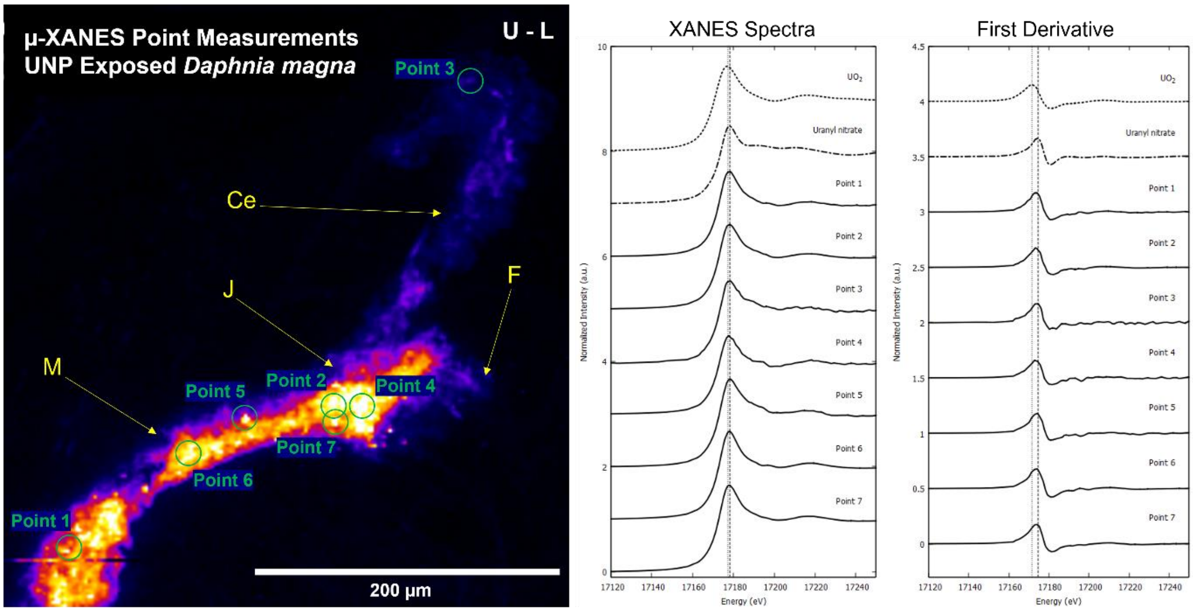

Figure S4: Point μ-XANES measurements in *D. magna* following exposure to UNPs (320 μg L^{-1}), selected after taking the μ-XRF image. Points 1, 5, and 6 were located away from the junction of the midgut and hepatic ceca, while points 2, 4, and 7 were at that junction immediately following the foregut. Point 3 was towards the anterior of the hepatic ceca. XANES spectra and the first derivative are presented together with U(IV) and U(VI) reference compounds (UO_2 and uranyl nitrate, respectively). The absorption edge is presented by the dashed line. *Abbreviations:* hepatic ceca (Ce), foregut (F), junction (J), midgut (M).

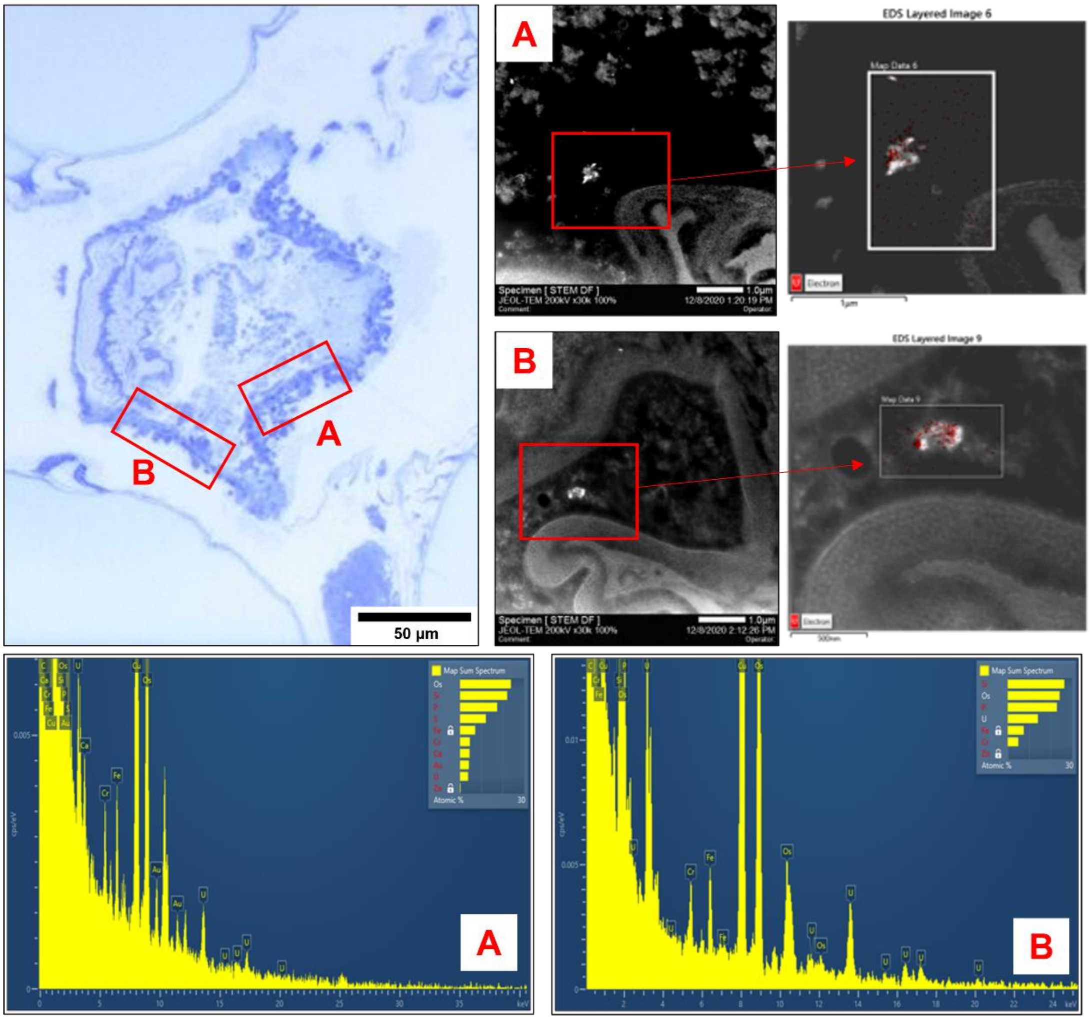

Figure S5: Transmission Electron Microscopy examinations of midgut sections, as indicated in the histology image, from UNP exposed *D. magna* (320 µg L^{-1}). Two investigation sites are noted where small (< 250 nm) high density aggregates were found along the brush border of the epithelial cells, as shown in the STEM images (A and B). Energy dispersive X-ray spectroscopy analysis shows the particles contain U, further supported by the EDS spectra on the left, indicating that UNP aggregates are present at least along the epithelial cells.
